# Fabry–Perot Resonance in 2D Dielectric Grating for Figure of Merit Enhancement in Refractive Index Sensing

**DOI:** 10.3390/s21154958

**Published:** 2021-07-21

**Authors:** Suejit Pechprasarn, Suvicha Sasivimolkul, Phitsini Suvarnaphaet

**Affiliations:** College of Biomedical Engineering, Rangsit University, Pathum Thani 12000, Thailand; suejit.p@rsu.ac.th (S.P.); suvicha.sa61@rsu.ac.th (S.S.)

**Keywords:** grating Fabry–Perot, 2D grating, optical sensors, optical resonators, sensor enhancement, instrumentation

## Abstract

We have recently reported in our previous work that one-dimensional dielectric grating can provide an open structure for Fabry–Perot mode excitation. The grating gaps allow the sample refractive index to fill up the grating spaces enabling the sample to perturb the Fabry–Perot mode resonant condition. Thus, the grating structure can be utilized as a refractive index sensor and provides convenient sample access from the open end of the grating with an enhanced figure of merit compared to the other thin-film technologies. Here, we demonstrate that 2D grating structures, such as rectangular pillars and circular pillars, can further enhance refractive index sensing performance. The refractive index theory for rectangular pillars and circular pillars are proposed and validated with rigorous coupled wave theory. An effective refractive index theory is proposed to simplify the 2D grating computation and accurately predict the Fabry–Perot mode positions. The 2D gratings have more grating space leading to a higher resonant condition perturbation and sensitivity. They also provide narrower Fabry–Perot mode reflectance dips leading to a 4.5 times figure of merit enhancement than the Fabry–Perot modes excited in the 1D grating. The performance comparison for thin-film technologies for refractive index sensing is also presented and discussed.

## 1. Introduction

In recent years, optical resonators [1,2] are one of the favored structures in sensors for sensing applications, such as biomedical sensing [3], refractive index sensing [4], and ultrasonic detection [5,6,7] due to their high-quality factor (Q factor) of the narrow resonant mode [8], which arises from resonant cavity [9].

**Figure 1:**
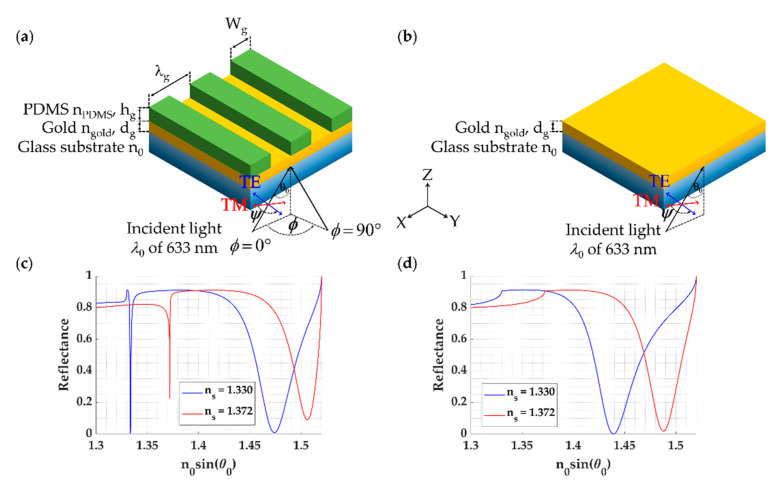
Schemes of (**a**) 1D rectangle grating for FP mode coupling; (**b**) Kretschmann configuration- based surface plasmon resonance structure; (**c**) Reflectance spectrum of 1D rectangle grating for FP mode coupling, and (**d**) Reflectance spectrum of surface plasmon resonance configuration for sample refractive indices of 1.33 (water) and 1.372 (bovine serum albumin (BSA) protein solution) when the structures were illuminated by a linearly TM-polarized coherent laser source at 633 nm wavelength.

At present, there are several types of resonators, including thin-film resonators [10], ring resonators [11], and grating waveguides [12]. Our previous work [13] has identified that subwavelength and near wavelength dielectric grating can serve as Fabry–Perot (FP) resonant cavity. The FP resonances excited in a thin film-based grating consisting of a thin gold layer and a one-dimensional (1D) rectangular dielectric grating, as shown in Figure 1a. The FP mode allows convenient sample access from the open space similar to surface plasmon resonance (SPR) detection [14,15,16] with a uniform gold layer of 48 nm as shown in Figure 1b, unlike well-known FP resonators, such as Bragg reflectors [8,17,18]. The dielectric grating is a lossless structure; the gold layer provides a loss mechanism [15,19] for the FP mode. Figure 1c shows reflectance spectra of 1D polydimethylsiloxane (PDMS) grating ($n_{PDMS}$ = 1.43 [20]) as depicted in Figure 1a with the grating thickness (*h_g_*) of 900 nm; grating period (*λ_g_*) of 791 nm; grating width (*W_g_*) of 237 nm, and a uniform gold layer with gold refractive index $n_{gold}$ of 0.18344 + 3.4332i [21]; the gold thickness (*d_g_*) of 48 nm and 38 nm for transverse magnetic (TM) polarization and transverse electric (TE) polarization, respectively, when the refractive index of the sensing region was water with the refractive index of 1.33 and bovine serum albumin (BSA) protein solution with the refractive index of 1.372 [22]. Figure 1c showed the reflectance spectrum for the 1D PDMS grating when the grating was illuminated by a TM linearly polarized coherent laser source at 633 nm wavelength. The sample fills the grating gaps; this has enabled the sample refractive index to embed as a part of the FP grating resonant cavity. In other words, the external sample refractive index can perturb the FP grating resonant condition. This feature is not present in the other closed FP resonant structures, such as bimetallic grating [8,23] and Bragg mirrors [24,25,26]. Figure 1d shows reflectance spectra of the SPR platform with the two refractive indices. Note that Figure 1c,d was calculated using rigorous coupled-wave theory; calculation details are provided in the Materials and Methods section below. Thus, both the SPR and the FP mode excited by the grating can respond to refractive index change in the same fashion.

In our previous work [13], we reported that the thin film-based grating resonator gave a narrow full-width at half maximum (FWHM) and better figure of merit (FOM), which leads to better refractive index sensing capability for small changes in the sample refractive index region. However, it has slightly lower sensitivity than the SPR, as shown in Figure 1c.

Having explained the sensing mechanism of the 1D FP grating mode, if more of the 1D grating dielectric material has been replaced with the sample, such as making narrower stripes or removing the material in the other direction making a 2D grating, these can lead to a higher sensitivity since there is more sample loaded inside the grating gap. The sensitivity is proportional to the volume of the gap region, and one may predict that the FOM can be enhanced by the same proportion only. This linear FOM enhancement is valid for 1D grating, but not the case for the 2D grating since the 2D grating can also enhance the Q factor or the FWHM leading to a further FOM enhancement.

This paper presents a theoretical framework for analyzing two-dimensional (2D) rectangular and circular dielectric gratings to enhance sensitivity and FWHM further, leading to a 4.5 times enhancement in FOM. Computation for 2D grating structures requires more resources and is time-demanding than those 1D gratings. An effective refractive index model for 2D gratings is proposed and discussed so that the complex optical diffractions and modes of the 2D gratings can be simulated by a uniform homogeneous layer of the effective refractive index to predict the responses of the 2D gratings with no need for extensive computing power [27,28]. The performance of optical structures for refractive index sensing reported in the literature is also quantified, compared, and discussed. To the best of the authors’ knowledge, analysis of Fabry–Perot Resonance in 2D grating structures has never been investigated and reported before in the literature.

## 2. Materials and Methods

### 2.1. 2D FP Grating Structures

**Figure 2:**
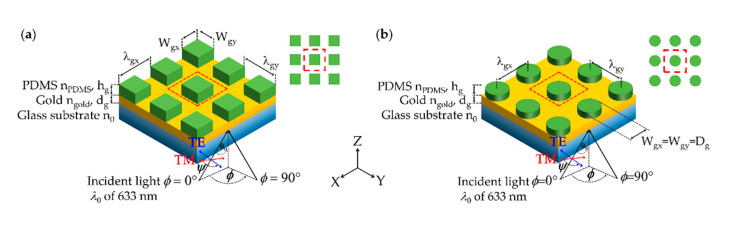
Schematic diagrams of (**a**) 2D rectangular pillar grating on a rectangular grid and (**b**) 2D circular pillar grating on a rectangular grid. The red dashed box indicates the unit cell for each scheme.

There are two 2D optical FP grating structures investigated here, which were:

(1) Rectangular pillars made of PDMS with $n_{PDMS}$ of 1.4283 [29] coated plasmonic gold sensor with thickness *d_g_* and refractive index $n_{gold}$ of 0.18344 + 3.4332i [21] on a standard BK7 glass coverslip with the refractive index *n*_0_ of 1.52. The rectangular grating was on a rectangular grid with the grating height of *h_g_*, the grating periods along the *x*-axis $\lambda_{gx}$ and *y*-axis $\lambda_{gy}$ with the width of deposited PDMS along the *x*-axis $W_{gx}$ and *y*-axis $W_{gy}$, respectively as shown in Figure 2a. The sensing region is on the top of the structure with the sample refractive index of *n_s_*. Grating fill factors along the *x*-axis *FF_x_* and the *y*-axis *FF_y_* are defined as *W_gx_/λ_gx_* and *W_gy_/λ_gy_*.
(2) Circular pillars made of PDMS with $n_{PDMS}$ of 1.4283 [29] coated plasmonic gold sensor with thickness *d_g_* and refractive index $n_{gold}$ of 0.18344 + 3.4332i [21] on a standard BK7 glass coverslip with the refractive index *n*_0_ of 1.52. The circular grating was on a rectangular grid with the grating height of *h_g_*, the grating periods along the *x*-axis $\lambda_{gx}\;$ and the *y*-axis $\lambda_{gy}$ are the same for the circular pillars. The diameter of deposited PDMS pillars is defined by *D_g_*, as shown in Figure 2b. The sensing region is on the top of the structure with the sample refractive index of *n_s_*. Grating fill factor along the *x*-axis *FF_x,_* and the *y*-axis *FF_y_* are the same and defined as *D_g_/λ_gx_* and *D_g_/λ_gy_*.

### 2.2. Optical Detection and Optical Simulation

The optical detection scheme measured the reflectance from the optical gratings when the gratings were illuminated by a linearly polarized coherent source with the incident wavelength $\lambda_{0}$ of 633 nm (Helium-Neon laser), the incident angle of *θ*_0_ in the glass substrate. The incident plane *ϕ* is defined relative to the *x*-axis and the polarization angle, *Ψ,* as depicted in Figure 2a,b. In this study, two linear polarizations were considered: the transverse magnetic (TM polarization or *p*-polarization) when was 0 rad, and the transverse electric (TE polarization or *s*-polarization) *Ψ* was π/2 rad.

Recently, in Sasivimolkul et al. [13], we have reported that the optimum gold thickness for the minimum reflectance dip was different for the TM polarization and TE polarization due to the different loss of energy dissipation of each polarization [13]. Therefore, the optimum gold thicknesses for TM polarization and TE polarization *d_g_* of 48 nm and 38 nm, respectively, were employed in this study.

Rigorous coupled-wave analysis (RCWA) [30,31,32] is employed to compute optical responses, including reflection coefficients, reflectance, and field distribution for the two types of the 2D gratings, as shown in Figure 2. The RCWA software was developed in-house under MATLAB environment utilizing parallel computing and graphic processing unit computing. All the simulations reported here were computed using diffracted orders in the *x*-axis and the *y*-axis of 21 orders and 21 orders, respectively, corresponding to the total diffracted orders of 441 orders to ensure that the simulation convergence has been reached.

### 2.3. Quantitative Performance Parameters

**Figure 3:**
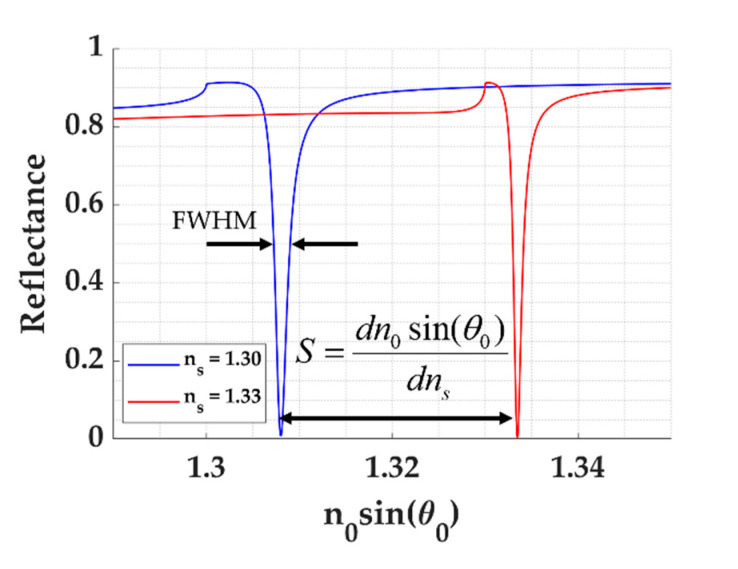
Shows how sensitivity and FWHM are calculated from the reflectance spectra.

Sensitivity (S) is defined as the change in the *n*_0_*sinƟ*_0_ over the change in sample refractive index *n_s_* as expressed in Equation (1) and shown in Figure 3. The sensitivity is defined using the change in the normalized wave-vector to reflect the coupling resonant condition of the FP and SP modes explained later in Equation (3). The *n*_0_*sinƟ*_0_ term can visualize the critical angle of the optical structure, allowing a direct comparison of refractive indices. It also indicates the numerical aperture (NA) required to excite the proposed FP modes. The change in *n*_0_*sinƟ*_0_ can be measured using back focal plane imaging [8,14,19,31], enabling simultaneous measurements of multiple modes through an objective lens. Other detection mechanisms, such as measuring the change in intensity level and measuring the change in the coupling wavelength, will be investigated and reported in a subsequent publication.

(1) $$S=\frac{dn_{0}sin\theta_{0}}{dn_{s}}$$

The full width at half maximum (FWHM) is defined as the width of reflectance dips at the intensity of 0.5, as depicted in Figure 3.

The figure of merit (FOM) is defined as the sensitivity divided by the full width at half maximum (FWHM) considering (1) how far the dip moves, which is the sensitivity, and (2) how narrow the dip is. The FOM is expressed as shown in Equation (2).

(2) $$FOM=\frac{S}{FWHM}$$

Dynamic range or detection range is defined as the range of sample refractive indices in which the sensor can still respond with the minimum reflectance dip of at least the reflectance of 0.25.

## 3. Results

### 3.1. Effective Refractive Index Model

**Figure 4:**
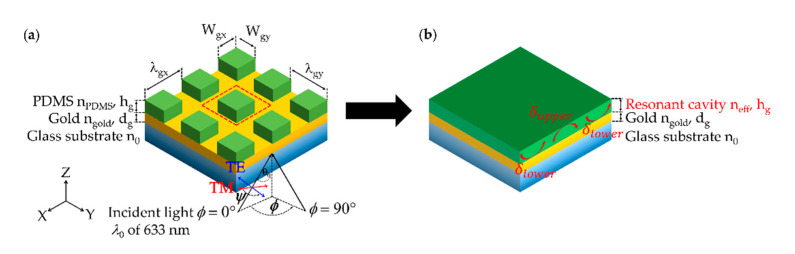
Shows (**a**) 2D rectangular pillar grating on a rectangular grid; (**b**) Effective refractive index model, and relevant parameters.

In Sasivimolkul et al. [13], we have recently reported an effective refractive index model that the FP mode position excited through 1D grating can be located using an asymmetric FP condition as expressed in Equation (3), where the grating layer can be simplified by a homogeneous layer of effective refractive index (*n_eff_*) and the layer thickness *h_g_* as depicted in Figure 4.

(3) $$2k_{z,cavity}h_{g}+\delta_{upper}+\delta_{lower}=2\pi M$$

The term $\delta_{upper}$ is the phase of reflection coefficient between the *n_eff_* layer and the sample layer. The term $\delta_{lower}$ is the phase of reflection coefficient between the *n_eff_* layer and the glass substrate layer. Note that the $\delta_{lower}$ and $\delta_{upper}$ can be calculated using Fresnel equations. *M* is the FP mode number, *M* = 0, 1, 2, ... It is established that the *M* of 0 only presents in TM polarization due to the existing TM polarization of a stripe line waveguide [33,34]. *k_z,cavity_* is the wave vector in the *z*-axis, which can be calculated as described by Equation (4). The difference between the 1D grating and the 2D grating is that the 2D grating diffracts light in both the *x* and *y* axes, whereas the 1D grating diffracts light in the *x*-axis only. In other words, the *K_y_* term in Equation (4) is 0 for 1D grating.

(4) $$k_{z,cavity}=\sqrt{{\left[\frac{2\pi }{\lambda_{0}}n_{eff}\right]}^{2}-{\left[K_{x}+K_{y}\right]}^{2}}$$

$K_{x}=k_{xi}+k_{g,x}$, $k_{xi}=\frac{2\pi }{\lambda_{0}}n_{0}\sin\theta_{0}\cos\phi$ and $k_{g,x}=\frac{m\pi }{\lambda_{gx}}$, *m* = 0, ±1, ±2, ±3, …

$K_{y}=k_{yi}+k_{g,y}$, $k_{yi}=\frac{2\pi }{\lambda_{0}}n_{0}\sin\theta_{0}\sin\phi$ and $k_{g,y}=\frac{n\pi }{\lambda_{gy}}$, *n* = 0, ±1, ±2, ±3, …

#### 3.1.1. D Rectangular Grating

The *n_eff_* for the 1D grating can be expressed as shown in Equation (5) [13]. It is crucial to point out that this *n_eff_* equation allows us to calculate and predict the mode position with no need for grating simulation software. This simplified model, of course, has some limitations. In Sasivimolkul et al. [13], the effective refractive index theory has been justified by comparing the optical response from the effective refractive index layer and the rigorous-coupled wave theory. The effective refractive index theory is valid for (1) the refractive index contrast between the two grating materials are less than 1, and (2) the grating period *λ_g_* is less than 2 times the optical incident wavelength, in which *λ_g_* is in subwavelength and near wavelength regimes. In other words, it does not consider the high index contrast grating behaviors, and it only calculates the response of the zeroth-order diffraction of the grating. The zeroth-order diffraction of rectangular grating has the highest diffraction energy in the subwavelength and near wavelength grating period.

(5) $$n_{eff}=\sqrt{n_{PDMS}^{2}FF+n_{s}^{2}\left(1-FF\right)}$$

**Figure 5:**
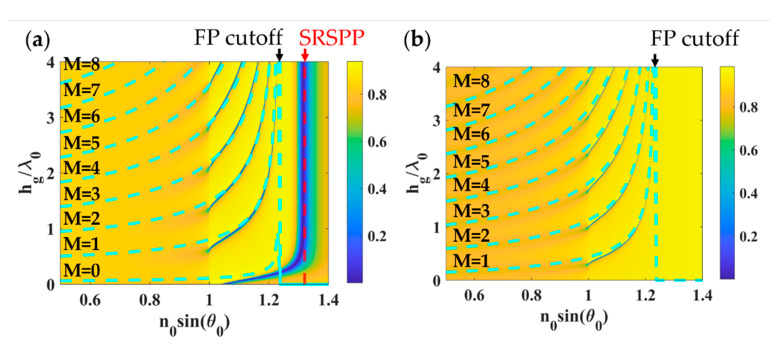
Shows results for 1D grating (**a**) TM polarization with 48 nm gold and (**b**) TE polarization with 38 nm gold. Other parameters: *h_g_* of 0 to 4*λ*_0_, *λ_g_* of 0.1*λ*_0,_ and *FF* of 0.5.

Figure 5 shows calculated FP mode positions using asymmetric FP condition expressed in Equation (3) and effective refractive index *n_eff_* express in Equation (5) (dashed blue curves) in comparison with the optical reflectance calculated using RCWA for the 1D grating with varying *h_g_* from 0 to 4*λ*_0_, *λ_g_* of 0.1*λ*_0_, *FF* of 0.5 and *d_g_* of 48 nm and 38 nm for TM polarization and TE polarization, respectively. It is clear to see that the proposed effective refractive index model can accurately predict the FP mode positions of the 1D grating. The red dashed curve shows the short-range surface plasmon polaritons (SRSPP) wave-vector $k_{SRSPP}$ labeled as ’SRSPP’ in Figure 5a, which can be approximated by surface plasmon dispersion relation [13], given by $2\pi /\lambda_{0}\sqrt{n_{gold}^{2}n_{eff}^{2}/\left(n_{gold}^{2}+n_{eff}^{2}\right)}$. 

#### 3.1.2. D Gratings

The 2D gratings can also be treated as a uniform dielectric layer with the layer thickness *h_g_* and effective refractive index (*n_eff_*). However, it is not as simple as a geometric averaging effect described in Equation (5). The *n_eff_* model for the 2D gratings also depends on the grating shape and the two-dimensional geometry. The *n_eff_* for the 2D gratings for different pillar shapes, including rectangular and circular pillars, can be expressed in Table 1.

**Figure 6:**
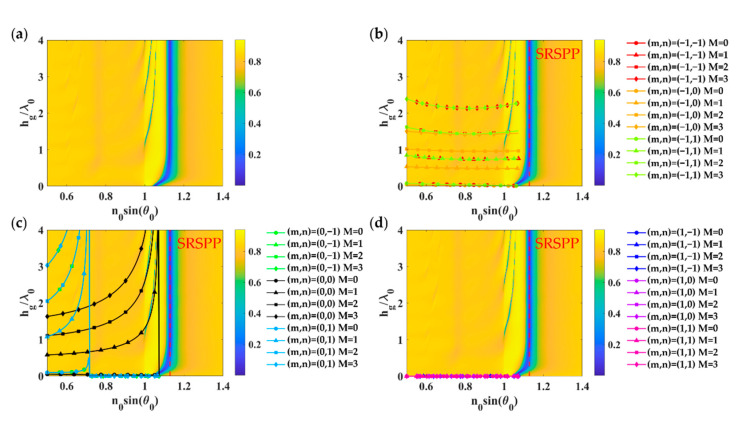
Optical responses of 2D rectangular grating with *FF_x_* and *FF_y_* of 0.5 (equivalent to $D_{g}$ of 0.625*λ*_0_), *λ_gx_* and *λ_gy_* of 1.25*λ*_0_ deposited on a uniform gold layer with the gold thickness *d_m_* of 48 nm when illuminated by linearly polarized TM wave at 633 nm wavelength: (**a**) Optical reflectance calculated using RCWA; (**b**) Grating diffracted orders *m* of −1; (**c**) Grating diffracted orders *m* of 0, and (**d**) Grating diffracted orders *m* of 1, with *n* of −1 to 1 for the FP mode numbers of 0 to 3 calculated using the effective refractive index model expressed in Equation (6) and the asymmetrical FP condition in Equation (3).

Figure 6a shows optical reflectance calculated using RCWA for the 2D rectangular grating with *FF_x_* of 0.3, *FF_y_* of 0.5, *λ_gx_*, and *λ_gy_* of 1.25*λ*_0_ deposited on a uniform gold layer with the gold thickness *d_m_* of 48 nm when illuminated by linearly polarized TM wave at 633 nm wavelength. There are FP modes after the critical angle of 1. The first mode that appears at the lower grating height is the short-range surface plasmon polaritons (SRSPP) labeled as ‘SRSPP’ in Figure 6b,d. Figure 6b shows different grating diffracted orders from *m* of −1 and *n* of −1 to 1 for the FP mode numbers of 0 to 3 calculated using the effective refractive index model expressed in Equation (6) and the asymmetrical FP condition Equation (3). Figure 6c,d was calculated using the same method as Figure 6b; however, they were calculated for *m* of 0 and 1 instead. Thus, the FP modes in the 2D grating were excited by the *m* of 0 and *n* of 0 diffracted order.

**Figure 7:**
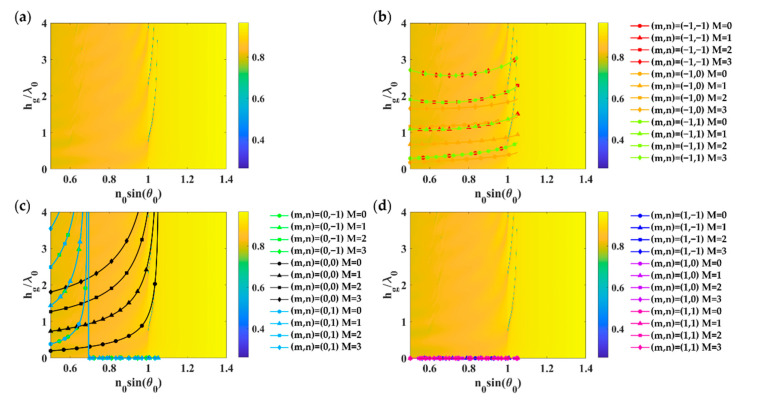
Optical responses of 2D rectangular grating with *FF_x_* of 0.3, *FF_y_* of 0.5, *λ_gx,_* and *λ_gy_* of 1.25*λ*_0_ deposited on a uniform gold layer with the gold thickness *d_m_* of 38 nm when illuminated by linearly polarized TE wave at 633 nm wavelength: (**a**) Optical reflectance calculated using RCWA; (**b**) Grating diffracted orders *m* of −1; (**c**) Grating diffracted orders *m* of 0, and (**d**) Grating diffracted orders *m* of 1, with *n* of −1 to 1 for the FP mode numbers of 0 to 3 calculated using the effective refractive index model expressed in Equation (7) and the asymmetrical FP condition in Equation (3).

Figure 7 shows the grating parameters’ response on a uniform gold thickness of 38 nm when illuminated by linearly polarized TM wave at 633 nm wavelength. Similar to the TM case, the refractive index model described in Equation (7) and the asymmetrical FP condition in Equation (3) can accurately predict the FP mode positions. The dominant FP modes were excited by the *m* of 0 and *n* of 0 diffracted order.

**Figure 8:**
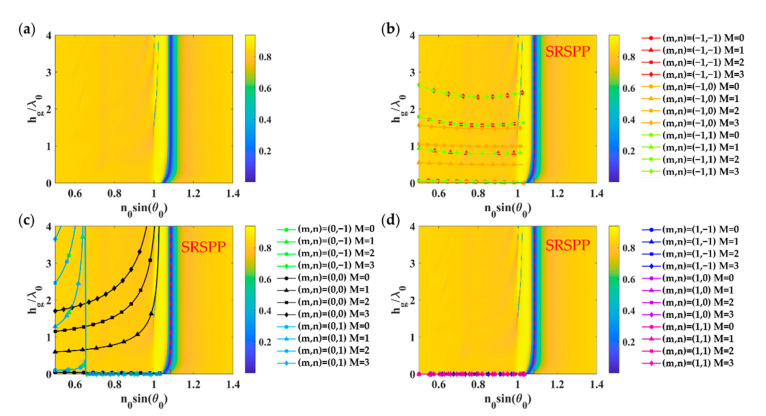
Optical responses of 2D circular grating with *FF_x_* of 0.3, *FF_y_* of 0.5, *λ_gx_*, and *λ_gy_* of 1.25*λ*_0_ deposited on a uniform gold layer with the gold thickness *d_m_* of 48 nm when illuminated by linearly polarized TM wave at 633 nm wavelength: (**a**) Optical reflectance calculated using RCWA; (**b**) Grating diffracted orders *m* of −1; (**c**) Grating diffracted orders *m* of 0, and (**d**) Grating diffracted orders *m* of 1, with *n* of −1 to 1 for the FP mode numbers of 0 to 3 calculated using the effective refractive index model expressed in Equation (8) and the asymmetrical FP condition in Equation (3).

**Figure 9:**
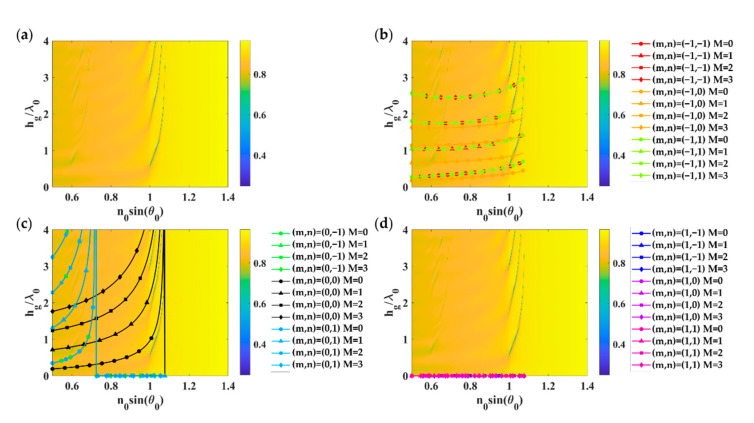
Optical responses of 2D circular grating with *FF_x_* and *FF_y_* of 0.5 (equivalent to $D_{g}$ of 0.625*λ*_0_), *λ_gx_* and *λ_gy_* of 1.25*λ*_0_ deposited on a uniform gold layer with the gold thickness *d_m_* of 38 nm when illuminated by linearly polarized TE wave at 633 nm wavelength: (**a**) Optical reflectance calculated using RCWA; (**b**) Grating diffracted orders *m* of −1; (**c**) Grating diffracted orders *m* of 0, and (**d**) Grating diffracted orders *m* of 1, with *n* of −1 to 1 for the FP mode numbers of 0 to 3 calculated using the effective refractive index model expressed in Equation (9) and the asymmetrical FP condition in Equation (3).

Similar to the rectangular grating, the proposed effective index model for circular pillars given by Equations (8) and (9) for TM polarization and TE polarization, respectively, can also provide correct mode positions compared to the RCWA calculation as shown in Figure 8 and Figure 9. Figure 8 and Figure 9 were calculated for *FF_x_* and *FF_y_* of 0.5 (equivalent to $D_{g}$ of 0.625*λ*_0_), *λ_gx_*, and *λ_gy_* of 1.25*λ*_0_ deposited on a uniform gold layer with the gold thickness *d_m_* of 48 nm and 38 nm for TM polarization and TE polarization. 

### 3.2. Refractive Index Sensing Performance of 2D Gratings

Although the proposed effective refractive index theory can predict FP mode positions and can be employed to calculate sensitivity, the sensitivity parameter alone is not a complete story about quantifying the refractive index sensing performance. In other words, for sensing application, not only how far the dip moves but also how wide the dip is [35]. The narrowness of the resonant dip cannot be computed from the refractive index model. Therefore, in this section, the results were computed using the RCWA.

**Figure 10:**
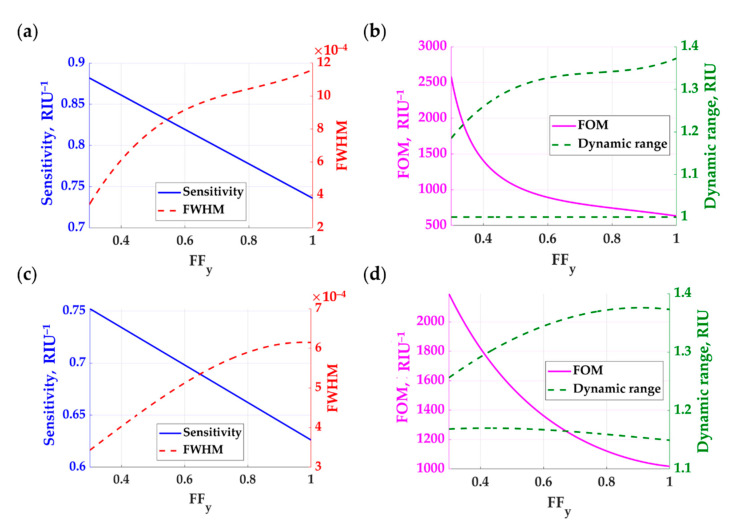
Refractive index performances of the 2D rectangular grating with varying *FF_y_* from 0.3 to 1, *h_g_* of 900 nm, *λ_gx_* of 1.25*λ*_0_, *λ_gy_* of 1.25*λ*_0_, *FF_x_* of 0.3, and varying *n*_s_ from 1.00 to 1.40 when a linearly polarized coherent light illuminated the gratings at 633 nm wavelength and the incident plane relative to the grating *ϕ* of 0 deg for (**a**) sensitivity and FWHM for TM polarization, **(b)** FOM and dynamic range for TM polarization, (**c**) sensitivity and FWHM for TE polarization, and (**d**) FOM and dynamic range for TE polarization. The sensitivity is shown in solid blue curves; the FWHM is shown in dashed red curves; the FOM is shown in solid pink curves, and the dynamic range is shown in dashed green curves.

Sasivimolkul et al. [13] reported that the 1D grating with *FF_x_* of 0.3, *h_g_* of 900 nm, and *λ_gx_* of 1.25*λ*_0_ is a practical sensor design, considering the fabrication feasibility and the proposed 1D grating does not show a strong mode hybridization. Hybridization between modes may obscure the continuity of FP dip movement, degrading the sensitivity and the detection range. Here, we have taken the 1D feature forward and remove the grating material along the *y*-axis, making a 2D grating structure by keeping the other parameters. Figure 10a–c shows the sensitivity in the blue curve and the FWHM in the red curve. The sensitivity is linearly proportional to the volume of the gap region available to the sample refractive index. The lowest possible *FF_y_* is at *FF_y_* of 0.3 before the FP mode cutoff occurs. It is pretty straightforward to predict that the sensitivity is enhanced by the amount of sample material inside the grating. However, this does not show how obvious the 2D features of the grating enhance the FWHM. There is nonlinear FWHM behavior depending on the *FF_y_*. The 2D rectangular grating can provide a narrower FP dip than the 1D grating case (*FF_y_* of 1), as shown in Figure 10b–d, leading to a 3.8 and 2.4 FOM enhancement factor for TM polarization and TE polarization, respectively. The FOM enhancement, however, comes with a tradeoff in the detection range. The detection range for the TE polarization was narrower than the TM polarization. Therefore, the TE polarization is not suitable for gas sensing, as shown in Figure 10d, whereas gratings with *FF_y_* more than 0.6 are suitable for biological sensing for the two polarizations, as shown in Figure 10b–d.

**Figure 11:**
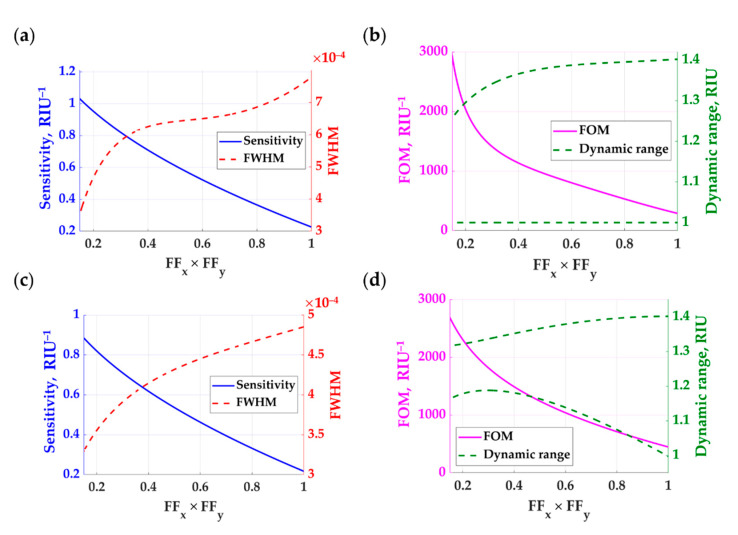
Refractive index performances of the 2D circular grating with varying *D_g_* from 300 nm to 800 nm, *h_g_* of 900 nm, *λ_gx_* and *λ_gy_* of 1.25*λ*_0_, and varying *n*_s_ from 1.00 to 1.40 when a linearly polarized coherent light illuminated the gratings at 633 nm wavelength and the incident plane relative to the grating *ϕ* of 0 deg for (**a**) sensitivity and FWHM for TM polarization, **(b)** FOM and dynamic range for TM polarization, (**c**) sensitivity and FWHM for TE polarization, and (**d**) FOM and dynamic range for TE polarization. The sensitivity is shown in solid blue curves; the FWHM is shown in dashed red curves; the FOM is shown in solid pink curves, and the dynamic range is shown in dashed green curves.

Figure 11a–c shows the sensing performance of circular pillar gratings with varying *D_g_* from 300 nm to 800 nm, *h_g_* of 900 nm, *λ_gx_* and *λ_gy_* of 1.25*λ*_0_, and varying *n*_s_ from 1.00 to 1.40 when a linearly polarized coherent light illuminated the gratings at 633 nm wavelength and the incident plane relative to the grating *ϕ* of 0 deg. The sensitivity for the $FF_{x}FF_{y}$ of 0.14 (equivalent to *D_g_* of 300 nm) was slightly higher than the rectangular grating with *FF_x_* and *FF_y_* of 0.3. Of course, the circular pillars allow the sample refractive index to fill up more space in the grating layer by the factor of $1-\pi \left(D_{g}^{2}/4\right)/\left(FF_{x}FF_{y}\right)$ equivalent to 21%. This extra sample space inside the grating accounts for the higher sensitivity of the circular pillar grating compared to the rectangular grating. The circular pillar grating also provided a narrower FWHM than the rectangular grating, as shown in Figure 11a–c compared to Figure 10a–c. These enhancements led to the FOM enhancement factors of 4.5 and 3.0 higher than the 1D grating for the TM polarization and TE polarization, respectively, as shown in Figure 11b–d. For the dynamic range, the rectangular grating has a similar detection range compared to the rectangular grating.

The performance parameters explained and defined in Section 2.3 are computed for the following thin film-based structures to make a direct comparison across different sensors:

(1) SPR sensor with 50 nm thick uniform gold layer coated on a uniform BK7 glass substrate with TM polarization illumination.
(2) Closed FP structure consisting of a sensing region sandwiched by two gold mirrors with the thickness of 45 nm and 90 nm [13].
(3) Closed FP structure consisting of a sensing region sandwiched by 2 Bragg mirrors with alternating refractive indices made of *n_low_* with the MgF_2_ refractive index of 1.37 [36] and *n_high_* with the TiO_2_ refractive index of 2.4 [37], and with the layer thickness of *λ*_0_/(4*n_low_*) and *λ*_0_/(4*n_high_*) for *n_low_* and *n_high_*, respectively. The Bragg mirror’s top and bottom consist of *n_high_, n_low_*_,_ *n_high_*, *n_low_*, and *n_high_* stacking.
(4) Open FP structure using 1D grating with *FF* of 0.3, *h_g_* of 900 nm, *λ_gx_* and *λ_gy_* of 1.25*λ*_0,_ and *d_g_* of 48 nm with TM polarization illumination.
(5) Open FP structure using 1D grating with *FF* of 0.3, *h_g_* of 900 nm, *λ_gx_* and *λ_gy_* of 1.25*λ*_0,_ and *d_g_* of 38 nm with TE polarization illumination.
(6) Open FP structure using 2D rectangular pillar grating with *FF_x_* and *FF_y_* of 0.3, *h_g_* of 900 nm, *λ_gx_* and *λ_gy_* of 1.25*λ*_0_, and *d_g_* of 48 nm with TM polarization illumination.
(7) Open FP structure using 2D rectangular pillar grating with *FF_x_* and *FF_y_* of 0.3, *h_g_* of 900 nm, *λ_gx_* and *λ_gy_* of 1.25*λ*_0_, and *d_g_* of 38 nm with TE polarization illumination.
(8) Open FP structure using 2D circular pillar grating with *D_g_* of 300 nm, *FF_x_* and *FF_y_* of 0.38, *h_g_* of 900 nm, *λ_gx_* and *λ_gy_* of 1.25*λ*_0_, and *d_g_* of 48 nm TM polarization illumination.
(9) Open FP structure using 2D circular pillar grating with *D_g_* of 300 nm, *FF_x_* and *FF_y_* of 0.38, *h_g_* of 900 nm, *λ_gx_* and *λ_gy_* of 1.25*λ*_0_, and *d_g_* of 38 nm TE polarization illumination.

Table 2 shows the refractive index sensing performance of the structures. For the sensitivity, the FP modes of the open grating structures were lower than the closed FP structures and slightly lower than the conventional SPR sensor. However, the FWHM for the open FP grating structures was two-fold narrower than the closed FP structures and the SPR measurement. The FWHM of the proposed 2D gratings were 2 to 3.5 times narrower than the previously reported 1D FP grating. The increase in sensitivity and narrower FWHM lead to a FOM enhancement of 4.5 times higher than the 1D FP grating and higher than the other compared thin-film structures.

## 4. Conclusions

The theoretical framework to analyze FP modes excited through subwavelength and near wavelength 1D and 2D grating structures has been proposed and discussed. The proposed open gratings allow convenient sample access from the top of the structure, such as surface plasmon resonance measurement. This feature is not present in the other closed FP grating structures, such as a spacer sandwiched by two metallic mirrors or Bragg mirrors. The grating layer provides a resonant cavity forming an FP mode. The grating gap is filled up by the sample leading to the FP resonant condition perturbation. The effective refractive index models for 1D grating, 2D rectangular pillars, and circular pillars have been introduced and verified by comparing the FP modes’ position excited through the gratings with reflectance calculated using rigorous coupled-wave analysis. The effective refractive index model allows the FP resonant mode condition to be computed with no need for time and resource-consuming 2D grating calculations. The limitations of the proposed effective refractive index model are (1) it is only valid for the grating index contrast less than 1, (2) it can only calculate the response from the 0th diffraction order. The 0th diffracted order has the highest strength for the proposed 1D and 2D gratings. For the sensing performance, the FP modes in the 2D gratings not only have higher sensitivity than the 1D grating since there is more sample material filling up the more significant grating gaps, but the FP dips also become narrower leading to 4.5 times enhancement in FOM compared to the FP modes in the 1D grating.

## Figures and Tables

**Table 1:** Effective refractive index equations for 2D gratings with rectangular pillars and circular pillars.

Structure	Effective Refractive Index	
Rectangle pillar		
TM polarization	$n_{eff}=\sqrt{n_{2}^{2}FF_{x}FF_{y}+n_{3}^{2}\left(1-FF_{x}FF_{y}\right)}$	(6)
TE polarization	$n_{eff}=\frac{1}{2}\sqrt{n_{2}^{2}FF_{x}FF_{y}+n_{3}^{2}\left(1-FF_{x}FF_{y}\right)}+\frac{1}{2}\sqrt{1/\left(\frac{FF_{x}FF_{y}}{n_{2}^{2}}+\frac{1-FF_{x}FF_{y}}{n_{3}^{2}}\right)}$	(7)
Circular pillar		
TM polarization	$n_{eff}=\sqrt{\frac{1}{\lambda_{gx}\lambda_{gy}}\left(n_{2}^{2}\pi {\left(\frac{D_{g}}{2}\right)}^{2}+n_{3}^{2}\left(\lambda_{gx}\lambda_{gy}-\pi {\left(\frac{D_{g}}{2}\right)}^{2}\right)\right)}$	(8)
TE polarization	$n_{eff}=\frac{1}{2}\sqrt{\frac{1}{\lambda_{gx}\lambda_{gy}}\left(n_{2}^{2}\pi {\left(\frac{D_{g}}{2}\right)}^{2}+n_{3}^{2}\left(\lambda_{gx}\lambda_{gy}-\pi {\left(\frac{D_{g}}{2}\right)}^{2}\right)\right)}+\frac{1}{2}\sqrt{\lambda_{gx}\lambda_{gy}/\left(\frac{\pi {\left(\frac{D_{g}}{2}\right)}^{2}}{n_{2}^{2}}+\frac{\left(\lambda_{gx}\lambda_{gy}-\pi {\left(\frac{D_{g}}{2}\right)}^{2}\right)}{n_{3}^{2}}\right)}$	(9)

Note that $D_{g}$ is the diameter of grating circular pillars.

**Table 2:** Performance of each structure in refractive index sensing.

Structure	Sensitivity, RIU^−1^	FWHM	FOM, RIU^−1^	Dynamic Range, RIU
Conventional SPR [13]	1.1870	0.0349	34	1.00–1.40
Two metallic [13]	9.0811	0.0248	365	1.23–1.36
Bragg mirrors [13]	13.0490	0.0307	426	1.10–1.27
1D grating with TM polarization [13]	0.7236	0.0011	680	1.00–1.38
1D grating with TE polarization [13]	0.6287	0.0006	902	1.00–1.35
Rectangular 2D grating with TM polarization	0.8820	0.0003	2580	1.00–1.18
Rectangular 2D grating with TE polarization	0.7522	0.0003	2190	1.17–1.26
Circular 2D grating with TM polarization	1.0298	0.0003	3040	1.00–1.25
Circular 2D grating with TE polarization	0.8902	0.0003	2720	1.16–1.32

Note that RIU stands for refractive index unit.

## Data Availability

Not applicable.
